# Immune regulation of host energy metabolism and periodicity of malaria parasites

**DOI:** 10.1098/rstb.2023.0511

**Published:** 2025-01-23

**Authors:** Isabella Cristina Hirako, Theresa Ramalho, Ricardo Tostes Gazzinelli

**Affiliations:** ^1^ Laboratory of Immunopathology - Instituto René Rachou, Fundação Oswaldo Cruz - Minas, Belo Horizonte 30190-002, Brazil; ^2^ Department of Molecular Cell and Cancer Biology, University of Massachusetts Chan Medical School, Worcester, MA 01605, USA; ^3^ Departamento de Bioquímica e Imunologia, Universidade Federal de Minas Gerais, Belo Horizonte, Brazil; ^4^ Centro de Tecnologia de Vacinas, Universidade Federal de Minas Gerais, Belo Horizonte, Brazil

**Keywords:** malaria, circadian cycle, *Plasmodium*

## Abstract

The synchronization of *Plasmodium* parasites as they replicate within red blood cells of their vertebrate host remains largely unexplored. Understanding this synchronization could reveal how parasites optimize their lifecycle to maximize transmission, evade the immune response and maximize energy acquisition. Rhythmic replication fulfils some criteria of an endogenous oscillator with time of day cues potentially provided by temperature, oxygen levels, hormones and/or nutrient availability. Recent research on a rodent malaria model has highlighted that rhythms associated with the host’s feeding/fasting cycle are a crucial factor influencing the synchronization of the erythrocytic stages of *Plasmodium* to the host’s circadian cycle. Innate immune responses are also rhythmic and can regulate host metabolism, suggesting that the innate immune response triggered by *Plasmodium* contributes to its rhythmic replication. Here, we outline how the interplay between immune responses and metabolism could influence the timing and synchronization of *Plasmodium*’s replication rhythm, focusing on the roles of the cytokine tumour necrosis factor, mitochondrial function and metabolites generated by the tricarboxylic acid cycle in highly activated monocytes. These processes are pivotal in controlling parasitemia and determining disease outcome, suggesting that a better understanding of energy metabolism on rhythmic host–parasite interactions may provide new insights for therapeutic interventions against malaria.

This article is part of the Theo Murphy meeting issue ‘Circadian rhythms in infection and immunity’.

## 1. *Plasmodium* infection and malaria

Malaria is a deadly infectious disease caused by a protozoan parasite belonging to the *Plasmodium* genus within the Plasmodiidae family, part of the Apicomplexa phylum. Alongside coronavirus disease 2019 (COVID-19), acquired immunodeficiency syndrome and tuberculosis, malaria ranks among the top four killer infectious diseases in the world. Globally, in 2022, there were an estimated 241 million malaria cases reported across 85 malaria-endemic countries, of which above 90% occurred in Africa [1]. While the last few decades have seen progress in reducing malaria cases, this has stalled, with a significant increase in malaria deaths in 2020–2022 compared to the previous year, with an estimated 627 000 deaths attributed to the disruption of essential malaria services during the COVID-19 pandemic [2].

The life cycle of malaria parasites in the vertebrate host commences with the bite of a female *Anopheles* mosquito, which introduces sporozoites along with saliva [3]. Upon entry into the human dermis, sporozoites migrate to the liver via the bloodstream. Sporozoites invade hepatocytes, and they undergo multiplication, each producing a multitude of merozoites. Upon release into circulation, merozoites invade red blood cells, developing within parasitophorous vacuoles. This developmental process encompasses three distinct stages: the ring stage, trophozoite stage and schizont stage. Following invasion, the merozoites promptly transform into the ring stage, progressing into trophozoites, which, upon maturation, initiate replication, forming schizonts composed of merozoites. Throughout most species of *Plasmodium*, individual parasite cells develop into rings, trophozoites and schizonts in synchrony and transition between these stages at certain times of day [4]. Upon rupture of infected red blood cells, the newly formed merozoites are released into the bloodstream, leading to the reinvasion of red blood cells and the commencement of a new replicative cycle. This cyclic pattern of invasion and burst of infected red blood cells leads to exponential replication of the parasite and symptoms of disease, including periodic fever [5].

The first records of malaria were found in Chinese and Egyptian writings from 3000 years BC. Ancient religious and medical texts related acute fevers to the gods’ punishment and the presence of evil spirits. In the Hippocratic era, malaria was known simply as ‘the fever’. Hippocrates was the first to discard superstitions, describing the intermittent paroxysm (chills, fever and sweats), the tertian fever associated with splenomegaly [6,7]. In Ancient Greek writings, Hippocrates characterized the fever: ‘When the paroxysms fall on even days, the crises will be on even days; and when the paroxysms fall on odd days, the crises will be on odd days’ [7]. In 1880, the military physician Charles Laveran was the first person to identify parasites in the blood of patients infected with malaria. During his work in Algeria, he observed blood forms of the parasite that developed inside human red blood cells [8,9]. For discovering the etiological agent of malaria, Dr Laveran received the Nobel Prize in 1907.

In 1886, 6 years later, Camillo Golgi confirmed the findings of Laveran and elucidated the cycle of the *Plasmodium* in red blood cells and the temporal coincidence between recurrent chills and fever with the rupture and release of parasites into the bloodstream [10]. He formulated a malaria toxin hypothesis, proposing that toxin was released during the red blood cell rupture causing cyclical fever following the synchronous replication of the parasite [7,11]. Malaria-induced fever was applied by a professor of psychiatry and neurology in Vienna, Julius Wagner-Jaureg. Dr Wagner-Jaureg’s approach was to treat neurosyphilis mental illness by inoculating malaria parasites to raise body temperature high enough to kill the causative bacteria [12]. The malaria toxin hypothesis was validated by the discovery that components released during the synchronous rupture of erythrocytes strongly activate the innate inflammatory response and culminate in a massive release of pyrogenic cytokines, e.g. interleukin-1β (IL-1β) and tumour necrosis factor-α (TNF-α). Consequently, intervals between the peaks of paroxysms coincide with the duration of the intraerythrocytic development cycle of *Plasmodium* infection [5,13,14]. He was awarded the Nobel Prize in 1927 for developing a method to treat paralytic dementia (an advanced stage of neurosyphilis) by inoculating malaria parasites into patients. He noted that approximately 70% of the individuals who developed high fevers were cured of syphilis. The pyrotherapy was used from 1917 to the mid-1940s, before the discovery of penicillin, employing *Plasmodium vivax* infection, as it produced high and prolonged fevers but was not as lethal as *P. falciparum* [12].

## 2. The role of innate immunity in the pathogenesis of malaria

Decades of research have provided an extensive understanding that the pathogenesis of malaria involves the activation of innate immune receptors, which play a crucial role in immune surveillance by detecting the presence of *Plasmodium* parasites and initiating protective immune responses [5]. However, the excessive activation of these receptors may also lead to a systemic inflammation and poor outcome of malaria [5,14]. These receptors, initially thought to be solely the Toll-like receptors (TLRs), are responsible for recognizing specific molecules from parasites and trigger the release of pro-inflammatory cytokines and initiate the immune response upon primary infection with microbial pathogens. However, over the last two decades, other families of innate immune receptors such as Nod-like receptors (NLRs) and inflammasomes as well as cyclic GMP-AMP synthase (cGAS), retinoic acid-inducible gene I receptor (RIGI) and C-type lectin receptors have also been implicated in sensing *Plasmodium* infection and been involved both in host resistance to infection and the pathogenesis of malaria [4,5,13,14].

In contrast to the antigen-specific receptors (T-cell receptors and membrane immunoglobulin) found on T and B lymphocytes, there are relatively few pattern recognition receptors (PRRs) that recognize highly conserved pathogen-associated molecular patterns (PAMPs) found in certain categories of pathogens across the microbial phylogenetic tree [15]. PAMPs are microbial structures recognized by different types of PRRs, including TLRs, NLRs, cGAS and RIGI. The distinction in the localization allows TLRs to recognize PAMPs at the endosomes or at the cell surface membrane, while mRNA and DNA sensors and NLRs are cytosolic receptors [16–19]. Multiple studies have revealed the important role of these PAMPs, such as glycosylphosphatidylinositol (GPI) anchors, hemozoin and immunostimulatory nucleic acids (DNA and RNA), in the initiation of the innate immune response against human parasites *P. falciparum* and *P. vivax* as well as the animal model species of *Plasmodium* that infects mice [5,20–27].

The activation of innate immune cells and subsequent systemic inflammation are key factors in the initial manifestations of malaria upon *Plasmodium* infection and influence whether severe disease develops [5,28]. The massive release of pro-inflammatory cytokines, the adhesion of infected red blood cells in capillary veins and the rupture and removal of infected red blood cells by splenic macrophages are correlated events that influence the development of major syndromes associated with malaria pathogenesis, including systemic inflammation, anaemia, jaundice, respiratory distress, metabolic acidosis, as well as cerebral and placental malaria [5,28]. If *P. falciparum* is left untreated, uncomplicated symptomatic malaria can quickly progress to a life-threatening disease, but individuals who are repeatedly infected develop natural acquired immunity, resulting in low parasitaemia and asymptomatic infection, without deleterious activation of innate immune cells [29].

Many parameters of the immune system, such as cytokine expression, immune cell trafficking and phagocytosis, have been shown to fluctuate in a daily fashion. Furthermore, the cells of the immune system themselves, including macrophages, natural killer cells and lymphocytes, contain intrinsic biological clocks that regulate their function, which may have important implications for health and disease [30–32]. This regulation likely reflects a balance between mounting an effective response at the optimal time, while minimizing immunopathology. Recent studies in mice have highlighted that disruption of the circadian cycle of hosts increases susceptibility to both viral and bacterial infections, including foodborne and airborne pathogens [30].

## 3. Host circadian rhythms and periodicity of *Plasmodium* replication

Circadian clocks regulate biological rhythms and play a significant role in coordinating processes within cells and tissues, and organism-wide [33]. By aligning their activities with external rhythms and temporally compartmentalizing internal processes, organisms are thought to maximize their survival and reproduction and optimize the use of energy [33]. Importantly, pathogens and microbiota, including parasites, are subject to the daily rhythms of their hosts. These rhythms include metabolic activity, energy sources, hormones and immune responses, which all influence host–pathogen interactions [30–32]. Clock genes in humans and other vertebrates are also involved in the regulation of the circadian timekeeping genes. The expression of clock genes, such as Period (Per1, Per2 and Per3), Cryptochrome (CRY1 and CRY2), Brain and muscle ARNT-like 1 (Bmal1) and Circadian locomotor output cycles kaput (Clock) demonstrates different patterns of response in relation to stimuli and signalling pathways. Importantly, disruption of these clock genes has a significant impact on various physiological processes and systems [33].

Host daily rhythms may have a significant impact on the behaviour and physiology of parasites that influences their ability to infect and reproduce. For instance, *Wuchereria bancrofti* exhibits a circadian rhythm in migration within the vertebrate host, in which vector-infective stages (microfilaria) are released in the bloodstream during the night, when the mosquito vectors that transmit the parasite to humans are most active [4,34,35]. Similarly, host and vector rhythms can affect *Plasmodium* replication and transmission. For example, knockout mice lacking *Bmal1* expression in GABAergic neurons, including those in the suprachiasmatic nucleus, exhibit non-rhythmic food intake, diminished hypoglycaemia at 6 am (ZT 23) and 12 pm (ZT 5), and disrupted synchrony of *Plasmodium chabaudi* in infected red blood cells [36]. Similarly, double knockout mice lacking both Per1 and Per2, which show a disrupted circadian cycle, also display disrupted synchrony of *P. chabaudi* cell cycle in red blood cells [37]. However, the parasite cell cycle is restored by generating a feeding/fasting rhythm by using time-restricted feeding protocols [37]. These outcomes underscore the significance of host behaviour in the synchrony and timing of *P. chabaudi* cell cycle in the vertebrate host. Whether evolutionary pressures driving malaria parasites to adapt to the host circadian cycle aim to enhance parasite transmission, evade the host immune response, respond to resource availability, or achieve all these benefits remains unknown.

## 4. Time keeping by *Plasmodium*

Periodicity in different parasite stages occurs across a range of taxonomic groups. The life-threatening disease sleeping sickness caused by *Trypanosoma brucei* causes disruption to the host’s circadian cycle, advancing the time of day of sleeping [38]. *T. brucei* also has a circadian cycle that drives metabolic rhythms in two different stages of the life cycle: mammalian bloodstream and insect procyclic forms [39,40]. These rhythmic patterns are influenced by the external environment, such as light–dark and feeding–fasting cycles, and the parasite’s endogenous oscillator that drives rhythmic expression of approximately 10% of genes in *T. brucei* in *in vitro* conditions. These genes are associated with regulating cellular metabolism pathways, coinciding with fluctuations in intracellular adenosine triphosphate concentration [41]. Similarly, the migration of microfilariae, described above, between the bloodstream and tissues is sensitive to the daily rhythm in the level of oxygen in the host’s blood. For example, microfilariae migrate from the pulmonary capillary barrier to peripheral blood when the level of oxygen in the pulmonary artery and vein drop below 55 mm Hg [35]. This drop occurs at night when human hosts are resting, providing a cue for the nocturnal biting activity of their mosquito vector. However, in regions without nocturnal vectors, such as Polynesia and parts of Southeast Asia, the migration rhythm of the parasite varies, which may be explained by a single, dominant mutation in an internal clock [34,42,43]. However, an endogenous oscillator in *Wuchereria* is yet to be found, and the parasite might simply respond directly to changes in blood oxygen tension (called a ‘just in time’ strategy).

Recent progress has been made in uncovering *Plasmodium* synchronization and timekeeping [4,44]. The rodent parasite, *P. chabaudi*, is considered the species whose infection dynamics in host and vector most closely resemble *P. falciparum*, the primary species that infects humans [45]. Additionally, a notable advantage is that *P. chabaudi’s* replication cycle lasts 24 h, which mirrors the host’s circadian rhythm, making it easier to correlate rhythmic phenotypes of parasite and host than for human-infecting *Plasmodium* whose cycles last 48 or 72 h. For example, 57% of *P. chabaudi* genes follow approximately 24 h (‘daily’) rhythms in expression, and half of these genes lose their transcription rhythm when the parasite replicates 12 h out of synchrony with host rhythms [46]. This loss of gene expression rhythmicity could disrupt the essential process of different replication stages, particularly trophozoites, which engage in vigorous replication, leading to the accumulation of biological mass and consequently, a heightened metabolic ratio. Rijo-Ferreira *et al*. [47] provide further support for an intrinsic clock in *P. chabaudi*, revealing that in hosts housed in an arrhythmic environment (constant darkness) and mutant hosts with disrupted circadian clocks, rhythmic replication is maintained for several cycles. As is typical for non-model systems, identifying clock genes is extremely challenging, although an E-box DNA motif that is a binding site of CLOCK:BMAL1, the transcriptional activator complex in the core circadian clock mechanism in mammals, might be part of *Plasmodium’s* intrinsic oscillator [47,48].

## 5. Rhythmic in-host drivers of *Plasmodium* periodicity

### (a) Fever

IL-1β was first discovered for its property as a pyrogenic cytokine (termed leukocytic or endogenous pyrogen). TNF is pyrogenic, but a concentration of at least 20−50 times higher is needed to produce the same fever as 10 ngkg^-1^ IL-1β [49–51]. Importantly, both IL-1β and TNF-α are pyrogenic cytokines produced in high levels by phagocytes that are activated during *Plasmodium* infection, both in humans and mice [22,24,52–54]. The interaction of these pro-inflammatory cytokines with the anterior hypothalamus disrupts the normal thermoregulatory process. This disruption leads to an elevation in the set point of thermoregulation, causing fever [50]. IL-1β and TNF-α bind to receptors on hypothalamic neurons, triggering the synthesis of prostaglandin E2, which acts via the EP3 receptor to affect hypothalamic neurons that regulate thermoregulation, ultimately leading to the manifestation of fever [50,55].

*P. falciparum* loses synchrony in replication when maintained in *in vitro* cultures [56–58], suggesting they need a rhythmic time of day cue (or Zeitgeber) to regulate the timing and synchrony of the replication rhythm. Early work suggests fever regulates *P. falciparum* synchrony because exposure to a temperature of 40°C inhibits parasite growth when erythrocytes are cultured *in vitro* [58,59]. Furthermore, initially asynchronous cultures synchronize when subjected to a temperature of 40°C on alternate days, simulating the 48 h fever cycle of *P. falciparum* malaria [60]. The febrile response damages developing schizonts, selectively favouring the survival of young progeny (merozoites ring stage). Thus, regular exposure to elevated temperatures, as occurs in natural infections, is likely capable of synchronizing *Plasmodium* replication.

However, during malaria, fever can significantly impact and disturb the normal diurnal rhythms, causing fluctuations in the patient’s body temperature ranging from 37 to 40°C. Thus, whether fever is a reliable time cue or synchronizing force throughout infections is unknown. Other open questions include what causes the parasite to replicate synchronously enough in the first place to elicit a strong fever response [4]. Furthermore, the involvement of other host rhythms is likely necessary to prevent parasites from replicating faster and faster in subsequent cycles (because slow-developing parasites are damaged by fever). Another puzzle is that mice in research institutions are kept at 20–22°C and do not exhibit fever in response to *Plasmodium* infection, yet the parasite still synchronizes to the host’s rhythm [4,61]. Therefore, recent research has considered other host factors that might be responsible for *Plasmodium*’*s* synchronized replication in the vertebrate host.

### (b) Melatonin

An important circadian pacemaker synthesized by the pineal gland in the brain is the hormone melatonin. In humans, melatonin peaks in the early hours of the dark period, regulating sleeping patterns and other circadian rhythms. Application of melatonin to *in vitro* cultures of *P. falciparum* can increase the number of schizonts [44,62]. Melatonin stimulates the release of intracellular calcium and cAMP signalling cascades in the parasite that are essential for the growth and differentiation of parasite developmental stages. This has led to the suggestion that melatonin synchronizes the replication cycle *in vitro* [63].

Supporting observations include that exposure of *P. chabaudi* or *P. falciparum* to melatonin accelerates the developmental process towards schizont and parasite proliferation [64]. Replication becomes less synchronous in hosts whose pineal glands are removed, but this can be ameliorated by administering melatonin injections at night [64]. However, certain inbred mouse strains, such as C57BL/6 and BALB/c, lack the last enzymatic step needed to synthesize melatonin, yet their parasites are synchronous [65]. In addition, rodents are nocturnal and, unlike humans, in CBA and C3H mice where melatonin synthesis occurs, melatonin peaks 2 h before dawn, around 5:00 am (ZT 22). Furthermore, melatonin is also an important antioxidant, and parasites nearing the end of the replication cycle are the most vulnerable to oxidative damage. Therefore, the potential mechanism through which melatonin regulates the synchrony and timing of schizogony requires further examination [65,66].

### (c) Feeding

Studies conducted by Hirako *et al*. [61], Prior *et al*. [67] and O’Donnell *et al* [68] add to the evidence that *Plasmodium*’s replication rhythm is coordinated with the host’s circadian rhythm. By altering the timing of the light–dark cycle and restricting food access to either the daytime or night-time, these studies were able to alter the timing and synchrony of *P. chabaudi*’s replication rhythm, even in hosts without circadian clocks. Specifically, in all conditions, the ring stage peaks are associated with the timing of torpor and hypoglycaemia of the host while it is in the fasting phase of its foraging rhythm. Furthermore, the elevated glucose levels in chemically induced diabetic mice (i.e. via pancreatic β-cell destruction) reduce the synchrony of replicating parasites. A recent study confirms that the intake of food *per se* into the host’s body does not influence the replication rhythm [47], supporting a role for a rhythm associated with the availability of nutrients in the blood [63,69]. Hence, these studies reveal that the synchrony and timing of the parasite’s replication rhythm is aligned with daily rhythms associated with the digestion and metabolism of food rather than the light schedule [4,61,67,70,71].

Our main hypothesis builds on the knowledge that *Plasmodium* spp. do not have the enzymes involved in gluconeogenesis, which are entirely dependent on host glucose as the immediate source of energy for replication and biomass acquisition [72]. Indeed, glucose *in vitro* restriction induces parasite stasis, whereas *P. falciparum* does not survive in the absence of glucose [73–75]. Indeed, during the intraerythrocytic stage, *P. falciparum* depends on glycolysis for ATP generation. Since it has no energy stores and lacks the machinery for gluconeogenesis, a continuous supply of glucose is necessary for the parasite’s growth and replication. Consistently, Saliba and colleagues demonstrated that when inhibiting glycolysis with 2-deoxy-D-glucose (2-DG) and 2-DG drugs, parasite growth is inhibited [74]. In addition to relying on the host for glucose provision, certain amino acids and vitamins are also essential for *Plasmodium* to complete replication. Some of these factors, to some extent, can be scavenged by the parasite as it digests haemoglobin during its development [76]. However, the amino acid isoleucine is absent in human haemoglobin and at a low frequency in murine haemoglobin and may also regulate the parasite’s replication rhythm [77]. Isoleucine may also serve as a time-of-day cue used by the parasite to align its replication rhythm to the availability of glucose, amino acids and other essential nutrients that are most abundant during the host’s active phase [77].

## 6. Immunometabolism as a regulator of *Plasmodium* periodicity

### (a) TNF-induced hypoglycaemia

Kwiatkowski *et al*. [78] investigated TNF levels in the plasma of Gambian children infected with *P. falciparum* and its correlation with schizont rupture. This study indicated that TNF release, akin to fever, is paroxysmal, with a massive cytokine release occurring when a critical number of schizonts rupture simultaneously, stimulating monocytes and contributing to severe malaria pathology [78]. Consistently, TNF has been identified as a pivotal cytokine governing glucose metabolism and host energy regulation, thereby influencing disease manifestations in a murine model of malaria [36,69].

The first link between host metabolism and immune responses was suggested by a reduction in synchrony of *P. chabaudi*’s replication rhythm in mice lacking the TNF receptor that display attenuated hypoglycaemia [61]. Similarly, observations support the hypothesis that parasite replication is augmented under conditions of elevated blood glucose levels in humans. First, re-feeding hospitalized patients in Africa often results in malaria attacks [79]. Second, a large case–control study reported that individuals with type 2 diabetes mellitus in Kumasi, Ghana, exhibited a higher susceptibility to *P. falciparum* infection [80]. However, it was hypothesized that this vulnerability was due to altered olfactory signals, including expiration, in diabetic individuals, potentially leading to increased mosquito attraction and subsequent transmission [81] rather than different within-host dynamics. Third, severe malaria in children is associated with insulin resistance as indicated by delayed glucose uptake [82]. These different patterns of disease progression could be associated with rhythms in food intake and immunometabolism.

Studies performed in our laboratory and elsewhere indicate that during *Plasmodium* infection, in both humans and murine models, activated monocytes show enhanced glucose uptake, increased mitochondrial and tricarboxylic acid cycle activities, and upregulated expression of pro-inflammatory genes [21,36,61,83,84]. Our findings are aligned with those from Kwiatkowski *et al*. [78], by revealing a peak of TNF levels in the mouse blood after midnight, when the synchronized rupture of red blood cells containing schizonts occurs [61]. Furthermore, TNF signalling amplifies glucose uptake by hepatic and splenic monocytes while concurrently suppressing physical activity, food consumption, energy expenditure and clinical symptoms of disease [36]. This increased glucose metabolic rate and altered energy metabolism coincide with hypoglycaemia in the early hours of the host’s resting phase, which is accompanied by a predominance of the low-energy-consuming *Plasmodium* stage (rings and early trophozoites) within infected red blood cells [61]. The correlations between immune cell metabolism, host feeding/fasting and parasite developmental stage suggest that parasites are constrained in their development and biomass accumulation during the host’s resting phase, so they remain as rings stages.

**Figure 1.**
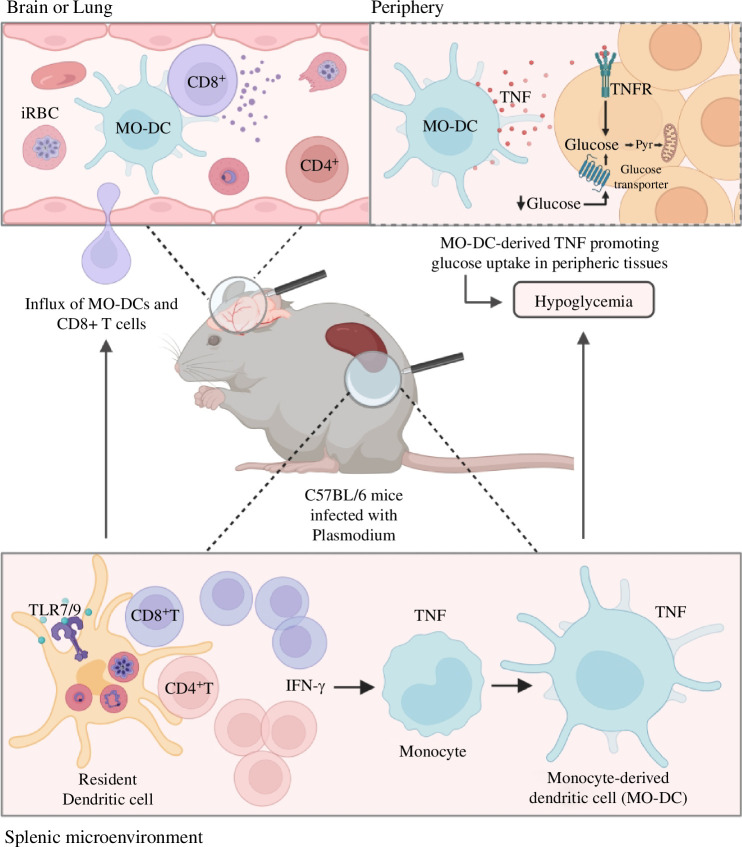
MO-DCs contribute to peripheral glucose consumption, which controls the rhythm of parasite proliferation in acutely infected mice. Infection with *Plasmodium berghei* ANKA strain leads to an IFN-γ-dependent differentiation of inflammatory monocytes into splenic MO-DCs (lower insert). Once differentiated, these MO-DCs migrate to the brain in response to CCR5 ligands, recruiting CD8^+^ T cells, which promote the development of experimental cerebral malaria (insert in the top left). In addition, in mice infected with *P. berghei* NK65 differentiated Tip-DCs emerge in the lung in a CCR4-dependent manner and mediate acute respiratory distress syndrome (ARDS). Finally, in *P. chabaudi*-infected mice, MO-DCs produce high amounts of TNF that affect various rhythmic parameters of host energy metabolism, such as physical activity, food intake, energy expenditure and respiratory exchange.

We also found that monocyte-derived dendritic cells (MO-DCs), both in the liver and spleen, are main up-takers of glucose during the systemic inflammation elicited by *Plasmodium* infection in mice [36]. Furthermore, this enhanced glucose uptake and metabolism by MO-DCs is mediated by interferon-gamma (IFNγ)/TNF-induced expression of glucose transporter 1 [21,36]. We hypothesize that while the host immune response is not exclusively responsible for synchronization of *Plasmodium*’s replication rhythm, it plays an important role by promoting glucose uptake by MO-DCs, and thus, lowering circulating glucose in a TNF-dependent manner. Several studies have reported that diet restriction limits parasite replication, attenuating disease in malaria mouse models [70,71]. MO-DCs are also involved in the pathogenesis of other experimental models of malaria, such as acute respiratory distress syndrome. These cells are high producers of TNF and contribute to peripheral glucose consumption, which controls the rhythm of parasite proliferation in acutely infected mice (figure 1).

## 7. Concluding remarks

The erythrocytic stage of *P. chabaudi* has a 24 h cell cycle that is aligned with the host circadian cycle. We emphasize how *P. chabaudi* synchronization is primarily determined by host cues, rather than an intrinsic clock of the parasite on its own. In particular, that parasite cell cycle is aligned with the daily rhythms of host feeding. We hypothesize that parasite dormancy, differentiation and replication are aligned with nutrient availability during the 24 h period of the host. Because *Plasmodium* parasites are entirely dependent on host glucose, we conjecture that raising glucose levels in the blood soon after feeding allows parasite replication when the host is hypoglycaemic. Intriguingly, we speculate that the inflammatory response elicited by the parasite also has a central role in this process, as TNF is an important regulator of host energy metabolism and promotes hypoglycaemia during acute infection with *P. chabaudi*. Hence, a better understanding of the role of glucose metabolism on the host–parasite interface may provide new insights for therapeutic intervention during malaria.

## Data Availability

This article has no additional data.
